# Quality of Life among Mothers of High Functioning Autism Spectrum Disorder (HFASD)Adolescents

**DOI:** 10.3390/ijerph19116663

**Published:** 2022-05-30

**Authors:** Nur Saadah Mohamad Aun, Siti Marziah Zakaria, Abdul Rahman Ahmad Badayai, Idayu Badilla Idris, Tuti Iryani Mohd Daud, Shima Dyana Mohd Fazree

**Affiliations:** 1 Centre for Research in Psychology and Human Well-Being, Faculty of Social Sciences and Humanities, The National University of Malaysia, Bangi 43600, Malaysia; marziah@ukm.edu.my (S.M.Z.); arab5487@ukm.edu.my (A.R.A.B.); p110731@siswa.ukm.edu.my (S.D.M.F.); 2 Department of Community Health, Faculty of Medicine, Universiti Kebangsaan Malaysia, Jalan Yaacob Latif, Bandar Tun Razak, Cheras, Kuala Lumpur 56000, Malaysia; idayubadilla.idris@ukm.edu.my; 3 Department of Psychiatry, Faculty of Medicine, Universiti Kebangsaan Malaysia, Jalan Yaacob Latif, Bandar Tun Razak, Cheras, Kuala Lumpur 56000, Malaysia; tutimd@ukm.edu.my

**Keywords:** quality of life, high functioning autism spectrum disorder, mothers, Malaysia

## Abstract

Autism Spectrum Disorder (ASD) has become more prevalent globally. The disorder is predominantly characterised by low social skills noted explicitly in people with High Functioning Autism Spectrum Disorder (HFASD). The individuals usually possess a normal or superior intelligence quotient (IQ) but the disability impedes the achievement of their actual high potential, hence compromising their quality of life (QoL). Managing adversities encountered by children with HFASD often compromises the QoL of the entire family. Thus, this study aimed to identify specific domains of QoL among mothers of high-functioning autistic adolescents. The study assessed seven mothers of adolescents with HFASD using a semi-structured interview format. A thematic qualitative analysis was conducted to analyse the data. The results suggested that mothers perceived their QoL based on physical and emotional well-being, material well-being, interpersonal relationship, and environmental well-being. Intervention for HFASD is multidisciplinary, which targets a broad spectrum of symptoms and skills deficits and customises the programme to meet each individual’s different needs. Nonetheless, intervention facilities in Malaysia are seriously limited, particularly in supporting QoL for children with HFASD. Therefore, by identifying the domains of QoL would improve the mothers’ resilience in raising their children with HFASD.

## 1. Introduction

The Autism Spectrum Disorders (ASD) are complex neurodevelopmental disorders with long-term effects on social communication and repetitive and restrictive behaviour patterns [1]. Autistic individuals hold varying degrees of intelligence; average or above-average intelligence is considered high functioning autism (HFA), while below-average intelligence is considered low functioning autism [2]. Although all individuals meet the core diagnostic criteria, functional estimates remain highly variable. Meanwhile, HFA is a term used to describe people with ASD with an IQ of 70 or higher [3]. Children and adolescents with HFA exhibit social competence heterogeneity, particularly noticeable during interactions with unfamiliar peers [4]. Autistic individuals reported more impediments and mental health concerns than their non-autistic peers. Despite their actual high potential, autistic youths have low QoL [5,6], specifically in the QoL social domain [7,8]. Social skills deficit is one of the primary deficits in HFASD that is significantly associated with low QoL. Significant challenges among autistic youths involve social aspects, such as social skills, social support opportunities, and ASD awareness levels of others [9]. Numerous research has demonstrated that parenting autistic children is a demanding situation, thus increasing the risk of parental stress and significantly reducing their well-being [10,11]. Parents frequently sacrifice their personal needs to prioritise their children’s needs. Consequently, parents experience high levels of concern and insecurity about their autistic child’s future from the moment they are diagnosed [12].

Considering that ASD has no cure, intervention is essential to improve prognosis [13]. Some autistic people claim they do not feel a need to socialise, or they hold negative perceptions of socialising based on experience (being bullied), or they socialise in ways that others do not recognise or understand [14]. Furthermore, autistic adults are often unemployed or underemployed due to their abilities and qualifications [15].

Cognitive Behavioural Therapy (CBT) intervention to teach autistic youths social skills is evidence-based and has proven to ultimately improve their QoL [16]. The significant positive predictors of QoL are from the social domain, including receiving support and being in a relationship [6]. The QoL refers to an individual’s satisfaction with their present life by considering their experiences and living conditions [17]. Satisfaction with their life will improve their social skills [18].

The World Health Organisation defined QoL as an individual’s “perception of their position in life in the context of the culture and value systems in which they live and in relation to their goals, expectations, standards, and concerns” [19]. The QoL is closely related to human rights and comprises health, well-being, living conditions, family relations, play, social life, education, and leisure. The QoL assessment for people with specific disabilities, such as ASD, is to facilitate their daily participation in the community.

The QoL is a multifaceted notion involving an individual’s view of their life and everyday activities [20]. The QoL is also a multidimensional construct measuring various domains, such as personal development, self-determination, interpersonal relations, social inclusion, rights, emotional well-being, physical well-being, and material well-being [21]. The QoL of autistic individuals is generally associated with social skills. Social skills for autistic children teach discrete social skills, such as joining a group or maintaining a conversational topic [22]. Therefore, social skills are always related to social communication skills, which is one of the main psychological needs among autistic youths [23]. Deficient social skills seriously impact various aspects of life, particularly QoL.

Youth with HFASD are diagnosed with ASD without intellectual disability and commonly graduate with regular high school diplomas [24]. Understanding the specific domains of QoL is crucial as this aspect is mostly affected by the social skills deficits among adolescents with HFASD [4], which will be targeted in therapy. The QoL among caregivers of individuals with mental disabilities, such as Attention-Deficit or Hyperactivity Disorder (ADHD) and ASD decreased during the pandemic [25].

Autistic children, adolescents, and young adults, including high functioning autistic individuals, have been reported to exhibit low QoL [26,27,28]. The situation is disappointing as they have high potential. Being in a healthy relationship is one significant predictor of QoL among autistic adults, while support for social difficulties will improve QoL [6]. Autistic children are challenged in various developmental domains, which is essential for their QoL. Apart from social communication skills, the study examined emotional well-being, social inclusion, interpersonal relationship, and parents’ perception. Generally, the domains are related to WHO’s definition of QoL, which includes a person’s physical health, psychological state, level of independence, social relationships, and relationship to the environment [29]. Social skills were linked with QoL among most autistic children [30]. Additionally, autistic young adults are at a high risk of poor QoL compared to those with other early-onset psychiatric and neurodevelopment disorders [31].

Parents of autistic children reported lower functioning of the overall family and their functioning as family members, while exhibiting higher levels of parenting stress and lower QoL [32]. Parent’s gender, marital status, education, family income, or perceived parental stress were predictors of QoL and the family income among families of autistic children [33]. Hence, the study aims to identify specific domains of QoL among mothers of high-functioning autistic adolescents.

## 2. Methods

### 2.1. Study Design

The philosophical orientation of this study is interpretivism, which this study was to understand the mother’s role in taking care of their HFASD child and improve their QoL. This is in line with interpretivism philosophy whom a mother plays as a social actor in explaining their experiences. The study employed a descriptive qualitative design based on a thematic analysis to explore the QoL among mothers of adolescents with HFASD. Furthermore, the study used a qualitative approach with an in-depth interview technique. The main research question was: how do mothers perceive their QoL in raising high-functioning autistic adolescents?

### 2.2. Participants

The study selected seven mothers as participants who fulfilled the following criteria: (1) the mothers of high-functioning autistic adolescents, (2) the mothers of HFASD children between the 12 to 21 years old, and whether they (3) consented or assented to participate in the study. The study exclusion criteria are (1) the adolescent has comorbid neurological disorders, including epilepsy and cerebral palsy, chromosomal disorders, or syndromic conditions based on their medical records. The criterion was set as to avoid bias and ensure that only those met the criteria would be included.

The study sample included participants within the age range from 41 to 55 years. The duration of diagnosis was from 15 months and between 1 to 36 months. All the adolescent participants attended mainstream kindergarten and moved to special needs schools for primary education. Four of the mothers are working, whereas three mothers are fully housewives. Regarding ethnicity, all seven mothers were Malay. Table 1 presents the participants’ characteristics.

### 2.3. Settings

Interview sessions were conducted in different locations. It is conducted based on the participants’ preferences. Geographically the location of the sessions were in Selangor and Perak, Malaysia.

### 2.4. Procedure

The study hired local professionals as the research team with expertise in psychology, psychiatry, and public health. The research team designed questions based on literature review and Quality of Life of Autism (QoLA) as an interview protocol to guide the in-depth interview session (see Table 2). The data collection commenced in early April 2021 until November 2021. After obtaining informed consent, interviews were audio-recorded and transcribed verbatim by the team’s research assistant. Subsequently, signed informed consent was obtained from each participant and in-depth interviews were conducted both online and face-to-face. The interview had to be performed online due to the Movement Control Order (MCO) following the coronavirus disease (COVID-19) outbreak.

The sessions were guided by the questions developed by the research team to explore QoL among mothers of HFASD adolescents. The interview session lasted approximately 60 to 90 min. Additionally, the questions were semi-structured to elicit participant information. The types of questions were open-ended, branching, and conversational (see Table 2). The participants were also asked to complete a short demographic survey, which comprised information about their children’s race, ethnicity, gender, and age. The participants were financially compensated with RM150.

### 2.5. Transcription and Data Analysis

Each interview was audio-recorded and transcribed verbatim by the research assistant. The researchers reviewed and transcribed data from the interview session. Analytic coding was used to identify themes and explore the QoL domains. Coders use analytical coding to identify themes and explore and develop categories and concepts from the transcribed data. Initially, open coding was performed to identify broad concepts in the data. Subsequently, selective coding was employed to thoroughly examine each conceptual category. Finally, a research team member analysed all codes and themes and divided them into conceptual categories. The validity of the results was confirmed through research team checking.

## 3. Results

The mothers discussed the QoL related to their child’s care during the interview. The themes that emerged from the interview responses linked to the mothers’ QoL comprised of these domains; (1) physical and emotional well-being, (2) interpersonal relationships, (3) material well-being, and (4) environmental well-being. The findings demonstrated the participants’ QoL with details as follows:

- Informant 1: A 50 year old executive working at a semi-government agency and a mother to a 13 year old boy with HFASD.
- Informant 2: A 41 year old housewife and a mother to a 14 year old boy with HFASD.
- Informant 3: A 44 year old working in a semi-government agency. A mother to 19 years old boy with HFASD.
- Informant 4: A 55 year old housewife and a mother to a 21 year old boy with HFASD.
- Informant 5: A 45 year old lecturer in a local university and a mother to an 18 year old boy with HFASD.
- Informant 6: A 50 year old teacher and a mother to a 20 year old boy with HFASD.
- Informant 7: A 54 year old teacher and a mother to a 21 year old boy with HFASD.

### 3.1. Physical and Emotional Well-Being

Most participants verified that their physical well-being was maintained throughout their lives. Moreover, they perceived excellent physical well-being over the years from the moment of diagnosis. The mothers also noted good physical health when receiving positive support from their spouses and children.

*“…Thank God… Luckily my husband and I are sport-person. Like my husband, he loves to play basketball… Every Monday and Wednesday I do HIIT, I join everything… Sometimes when we go for hiking, we bring him together… Put the picnic mat and he will play with his gadget…”*

Informant 1.

*“…Usually, my husband and he ride a bicycle together around this area… But since he scared of Covid… He stops ride his bicycle and no longer play football together with his friends… He is very restricted to the order…”*

Informant 2.

*“…I always enjoy doing Zumba with my friends… Usually I went for exercise after I chauffeur my kids to school… That is my free time, and I will make sure I have time for it…”*

Informant 4.

Parents’ concern over their children with disabilities was one of the significant factors that caused stress, specifically for mothers. Most mothers of high-functioning autistic adolescents stated that they are anxious about their children’s future. Some of their statements were as follows:

*“…If he goes out alone, we will worry about other things as well… Special kids like him is such a likeable. But people tend to take advantages on him…”*

Informant 6.

*“… He used to take a ride using train by his own… He went to the mall by himself… We never thought that he could be this brave… We are worries if he cannot go back home but surprisingly, he can remember his route to home… But we are still anxious about his future… I am scared if he cannot be independent enough… So, we ask our daughter to promise us to take care of her brother when we are gone…”*

Informant 3.

*“.. I just feel sad… Thinking about his future… What if I am gone too soon…? Who will take care of him like I do…?”*

Informant 4.

Whereas, only one mother worried about her child’s behaviour as he reached puberty. She said:

*“.. It just that we are worried about him… He started to watch some “adult” video… We are worried if he applies what he watched in reality… He used to show some “adult” pictures to his friends…”*

Informant 7.

### 3.2. Interpersonal Relationship

The participants agreed that the quality of friendship was more important than the quantity. With proper support received, the mothers felt more comfortable engaging in society.

#### 3.2.1. Social Support

The mothers discussed how acceptance by friends is vital. Receiving recognition from friends for raising an autistic child is one of the blessings.

*“…I always ignore what they say about my kid, and I always do not make friends with them. It’s better for me to keep a small group of friends who always support me”*

Informant 2.

*“…Many of my friends can’t believe that Dani is special because he looks normal. They are supportive. They are my officemates”*

Informant 1.

#### 3.2.2. Family Support

Most of the participants stated that family is an important supporter in raising adolescents with HFASD.

*“Not everyone can understand us, especially my kid, with this conditions. What I can do is to make sure that my kid had a support from me, and my husband is always here to support me”*

Informant 4.

*“I am blessed because my family is understand Dani’s conditions”*

Informant 1.

*“…I think all my family are very supportive… But when it comes to medication for my son, they are very judgmental… The doctor prescribed him with Ritalin… So all my siblings ask me to stop giving him the drugs…”*

Informant 3.

*“…I always share everything to my sibling… It makes me feel some kind of relief when I can share to them… But I do not share with my parents… I do not want them to feel worry…”*

Informant 2.

### 3.3. Material Well-Being

#### 3.3.1. Providing Accommodation

The mothers discussed that their material well-being is achieved when they can fulfil something requested by their autistic child without financial restraint. For instance, Informant 4 stated that:

*“…We provide him with all the luxury gameplay… We don’t mind how much we have to spend on his interest… As long as he’s happy then I’m happy… We don’t want him to play in an arcade, so we make his room like the arcade…”*

Informant 4.

#### 3.3.2. Access to Services

The mothers stated that they did not mind how far they must travel and how much they must spend on their children.

*“…We don’t really mind how much do we have to invest on him… We’ve registered a class for him like a culinary class under Mental Health Foundation… As long as he can learn something…*

Informant 3.

*“…I took him to Altuz Academy for his therapy… Even though it is expensive, but I don’t really mind… I’ve my own income and my husband is very supportive for each therapy that my kid are joining…”*

Informant 1.

*“I send my kid to private centre for his therapy… I don’t mind if I have to pay and travel to Ipoh because I can join the therapy and I can learn something…”*

Informant 6.

### 3.4. Environment Well-Being

#### 3.4.1. Safety

The mothers discussed the children’s safety awareness. Due to the child’s condition, all the mothers mentioned that their children did not engage in outdoor activities. Some of the statements were:

*“I don’t go anywhere and don’t give him to play outside. He understands that he has to keep safe. ”*

Informant 2.

*“I am very protective to him. I won’t let him go outside alone even in front of our home. But the children with Autism is lack of social skills, so, I don’t have to worry much because I know that he always at home”*

Informant 1.

*“I let him exercise around my home compound. He will just jog around. But I don’t let him go to the park. That is worrisome. ”*

Informant 4.

*“I will make sure my neighbor to look after him when I go to school… Just to ensure his safety. ”*

Informant 6.

#### 3.4.2. Stigma

When asked about the experience in raising autistic children, one mother stated experiencing stigma from different aspects.

*“I used to told my neighbors that my child is special kid… But they ignored… They asked their kids not to play with my child…”*

Informant 2.

There were mothers that stated that their child was asked to move to other schools despite their child obtaining good grades.

*“He used to go to normal kindergarten… But the teacher asked me to stop sending my child to the kindergarten… Because he is abnormal, and he can’t socialize…”*

Informant 6.

*“The teacher from the school terminate my child from taking any exam and asked me to change his school… Because they have to maintain the school’s performance and my child might jeopardize the school’s ranking…”*

Informant 5.

## 4. Discussion

The qualitative study used thematic analysis to identify QoL domains among mothers of adolescents with HFASD. The following section discusses key findings related to the outcomes and QoL for mothers with HFASD adolescents, possible explanations for the findings that diverge from past studies, the current study limitations, and implications for future research.

The physical health domain measures the parents’ energy level, daily activities, and sleep and work capacity [18]. The findings suggest that mothers cope very well in terms of physical health. Contrarily, past research demonstrated that parents with ASD children require extra caregiving demand [34]. Similar to other studies, physical and emotional well-being, interpersonal relationships, social support, family relations, material well-being, and environmental well-being were the QoL domains among mothers of adolescents with HFASD. The mothers’ concern for their children with disabilities was one of the main factors that stressed them. Furthermore, the presence of a child with disabilities poses irreparable outcomes to the family’s emotional well-being [35].

The availability of family and friends that can provide psychological and material resources minimises psychological distress [36]. Social support has been described as a critical resource for families with disabled children [37]. The type of support is one of the coping strategies for families who raise autistic children. The support received could lighten the stress and provide an insight for the parents to raise their special child. Many parents had experience receiving formal and informal social support they needed to educate their children [38]. Mothers of adolescents with ASD reported using a combination of formal and informal supports, which were perceived to be helpful for them [39]. Parents with autistic children perceive partners and families to provide greater support than friends and professionals [40].

Material well-being, such as monthly income, can unburden mothers’ emotional well-being in raising autistic children [41]. Material well-being also comprises access to transportation and medical care [42], including the aspect of feeling safe in the community [43]. The participants stated that they prioritise the healthcare services for their children regardless of financial concerns. Past studies highlighted that mothers had to spend considerable money to obtain treatment from NGOs or therapy [44].

Unsurprisingly, lack of speech production was the main indicator that led parents to seek a diagnosis for their children [45]. Parents’ culture and knowledge of ASD may impact the way they perceive autism and seek treatments for their children [46]. Although children require multiple services, families often encounter difficulty accessing and implementing recommended services [47].

Children with ASD often prefer solitary activities and many children favoured time spent alone to unwind and pursue their interests [48]. The child’s autism-related behaviours are strongly linked to isolation and exclusion, where the more such behaviours a child exhibits, the greater the family’s isolation and exclusion [49]. The findings present that the mothers experienced stigma in various circumstances. Notably, the stigma associated with ASD affects children and parents. Parents of autistic children are not immune to the influence of their co-workers’ opposing viewpoints in their workplace [50]. Nevertheless, some parents do not perceive this as a stigma. For example, a study discovered low scores on stigma and fair levels of stress and QoL, indicating that parents do not feel stigmatised by affiliation with an autistic child [51].

The results of the current study outlined that when the mothers of adolescents with HFASD are confronted with specific QoL, such as physical and emotional well-being, interpersonal relationships, material well-being, and environmental well-being.

The study presented strengths and limitations. First, the study performed an in-depth thematic analysis of the QoL of parents of HFASD youth. The assessment facilitated an understanding of the children’s physical and emotional well-being, material well-being, and environmental well-being. The data was collected through semi-structured interviews using triangulation between researchers to establish the main themes, which increased reliability.

Although the study sample was representative and provided sufficient information to examine the research question in-depth, the study had no large sample. Moreover, no triangulation was conducted with the study participants. The results have not been shared with them. Additionally, the study analysed the mothers’ experiences with HFASD children but did not consider the fathers’ QoL. Future multidisciplinary studies should consider the differences in the QoL of parents of HFASD youth based on different stages of symptomology and severity. Intervention and health facilities for HFASD should be improvised to mitigate the challenges for mothers in raising HFASD adolescents. Lastly, intervention and therapy might increase the QoL of adolescents with HFASD and improve the mothers’ QoL.

## 5. Conclusions

The study demonstrated that raising adolescents with HFASD is challenging, which involves difficulties providing education and formal skills to their children. The special adolescents require hands-on teaching, while the mothers need interpersonal relationships and family support. The unfulfilled needs will affect the mothers’ QoL. Hence, a better understanding of mothers’ QoL will encourage practitioners and researchers to enhance their understanding of mothers’ experiences and challenges in raising adolescents with HFASD. Thus, the study identifies specific QoL domains among mothers of adolescents with HFASD as an evidence base to plan interventions that can increase the QoL and improve the mothers’ resilience.

## Figures and Tables

**Table 1:** Sample characteristics.

Participants’ Names	Age	Employment Status	Health Issues	Adolescences’ Gender (Age)
Informant 1	50	Working	No	Boy (13 years)
Informant 2	41	Housewife	No	Boy (14 years)
Informant 3	44	Working	No	Boy (19 years)
Informant 4	55	Housewife	No	Boy (21 years)
Informant 5	45	Working	No	Boy (18 years)
Informant 6	50	Housewife	No	Boy (20 years)
Informant 7	54	Working	No	Boy (21 years)

**Table 2:** Protocol Interview.

No	Question
1.	Do you feel you are living a quality life? What are the quality things in your life?
2.	Tell us about your current state of health? Are you satisfied? How is your self-acceptance of your health?
3.	As we know, parents here are guardians to HFASD children. So, how do you know your child has HFASD? When did you realize there was such disorder?
4.	Your child has been diagnosed, so how does this treatment process take place?
5.	How is your acceptance of your child’s problems? What are your expectations for your child?
6.	How is the acceptance of a partner? Another child? Family? Friends?
7.	Tell us about the daily routine of your life? How to manage your daily activities?
8.	Who will help you manage family matters? What about your satisfaction with the help provided
9.	Are you satisfied with your sleep? Have you ever had trouble sleeping? If so, what are the steps to overcome sleep problems? What are the things that keep you from sleeping?
10.	What about your ability to perform the activities of your daily life? Managing children and family?
11.	Are you working? What about your ability to work? How do you divide your time between work and family management?
12.	Have you ever had negative feelings, such as sadness, frustration, anxiety or felt depressed? What causes these feelings? What are you going to do?
13.	What about opportunities for leisure activities? Do you still have time for recreation?
14.	Are you satisfied with the support you get from your friends? What support is provided?
15.	Are you satisfied with the conditions of your residence? Where do you live? What about the neighbors? Safety in the living environment?

## Data Availability

No new data were created or analyzed in this study. Data sharing is not applicable to this article.
